# New Opportunity to Formulate Intranasal Vaccines and Drug Delivery Systems Based on Chitosan

**DOI:** 10.3390/ijms21145016

**Published:** 2020-07-16

**Authors:** Roxana Popescu, Mihaela Violeta Ghica, Cristina-Elena Dinu-Pîrvu, Valentina Anuța, Dumitru Lupuliasa, Lăcrămioara Popa

**Affiliations:** 1 Department of Physical and Colloidal Chemistry, Faculty of Pharmacy, University of Medicine and Pharmacy “Carol Davila”, 020956 Bucharest, Romania; roxana_popescu@drd.umfcd.ro (R.P.); mihaela.ghica@umfcd.ro (M.V.G.); valentina.anuta@umfcd.ro (V.A.); lacramioara.popa@umfcd.ro (L.P.); 2 Department of Pharmaceutical Technology and Biopharmacy, Faculty of Pharmacy, University of Medicine and Pharmacy ”Carol Davila”, 020956 Bucharest, Romania; dumitru.lupuliasa@umfcd.ro

**Keywords:** chitosan, mucoadhesive, nasal pathway, nasal vaccines, nasal drug delivery systems

## Abstract

In an attempt to develop drug delivery systems that bypass the blood–brain barrier (BBB) and prevent liver and intestinal degradation, it was concluded that nasal medication meets these criteria and can be used for drugs that have these drawbacks. The aim of this review is to present the influence of the properties of chitosan and its derivatives (mucoadhesion, permeability enhancement, surface tension, and zeta potential) on the development of suitable nasal drug delivery systems and on the nasal bioavailability of various active pharmaceutical ingredients. Interactions between chitosan and proteins, lipids, antigens, and other molecules lead to complexes that have their own applications or to changing characteristics of the substances involved in the bond (conformational changes, increased stability or solubility, etc.). Chitosan and its derivatives have their own actions (antibacterial, antifungal, immunostimulant, antioxidant, etc.) and can be used as such or in combination with other molecules from the same class to achieve a synergistic effect. The applicability of the properties is set out in the second part of the paper, where nasal formulations based on chitosan are described (vaccines, hydrogels, nanoparticles, nanostructured lipid carriers (NLC), powders, emulsions, etc.).

## 1. Introduction

Polysaccharides have been extensively studied in recent years because of their numerous advantages, including their biocompatibility, biodegradability, good permeability, and a low price. Some examples of polysaccharides used in the biomedical field for the creation of innovative pharmaceutical products are starch, chitosan, cellulose, alginate, and caragheen [1].

**Figure 1:**
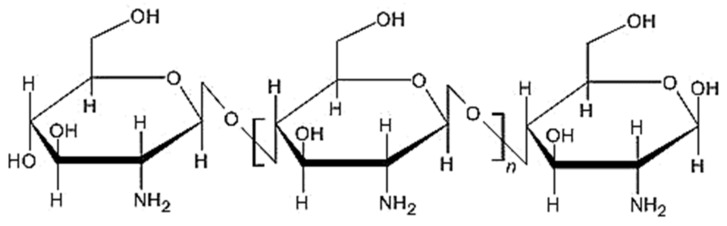
Structure of chitosan.

Chitosan, the second most common natural polysaccharide [1,2,3,4] and the only polycationic polysaccharide extracted from bio sources, is obtained from chitin by *N*-deacetylation [5]. The sources of chitin are crustaceans (crabs and shrimps), cephalopods [2], cuttlefish [5], insects (spiders, beetles, and ants) [3,6], fungi [1,3,4], spores [6], protozoa [2], yeasts [7], and algae [2,6]. Chitosan is a polymeric polysaccharide [5] (Figure 1) with a linear structure [8], is unbranched [9] and flexible [5], and has a high nitrogen content [6]. It has a high adsorption capacity [10,11,12] and permeability [13], but has a low mechanical strength [14]. It presents several polymorphic forms that impact its solubility, porosity, particle size, shape, and bioavailability [9].

Chitosan has free amino and hydroxyl functional groups [1,3,4] that can be involved in several chemical reactions, including carboxylation, alkalinization, esterification, hydroxylation, and acetylation, thus forming chitosan derivatives, which have applications in the biomedical field [9,15]. Chitosan’s solubility in acetic acid may limit its applicability in certain areas, such as wound healing, because acetic acid possesses cytotoxicity [16]. In order to overcome some of its solubility problems while maintaining the favorable properties, water-soluble chitosan derivatives (such as esther hydroxybutyl chitosan) have also been studied [17]. The disadvantage of these derivatives is the toxicity that can occur during chemical reactions [18].

The highly reactive amino functional group [8] induces the cationic character of chitosan, allowing interactions with negatively charged molecules [2].

Biomedical investigations on chitosan and its derivatives have been performed due to their properties, including their biocompatibility, biodegradability, non-toxicity, low allergenic activity [19,20,21,22], mucoadhesion, permeability enhancement [23,24,25,26,27], antibacterial and antifungal properties [28,29], immunogenic character [8,17,30], hemostatic properties [2,8], antioxidant activity [9], and antitumor effect [9,31]. Chitosan has several applications in biomedical and related fields, being used as a matrix for cell development and wound healing [8] (skin wound healing [32] and corneal wound healing [7,33]), tissue engineering (bone tissue engineering [15,34], skin tissue engineering, and cartilage tissue engineering [35]), gene delivery [36,37,38], the development of biosensors [39,40], adjuvant for mucosal formulations [34,41], cosmetics [42], and food packaging [43,44]. It is also a very important component in drug delivery systems [9] and the delivery of vaccines [18]. Due to their functional groups, chitosan and its derivatives can be used as a matrix for gels [45,46,47,48], emulsions [49], nanofibers [50], microparticles [51], nanoparticles [52,53], and other formulations.

The topic of this article is based on the analysis of the properties of chitosan and its derivatives, as well as the relationship between them and the formulations based on chitosan with nasal administration.

## 2. Properties of Chitosan and Its Derivatives

### 2.1. The Mucoadhesive Action and Permeability Enhancer Effect of Chitosan

**Figure 2:**
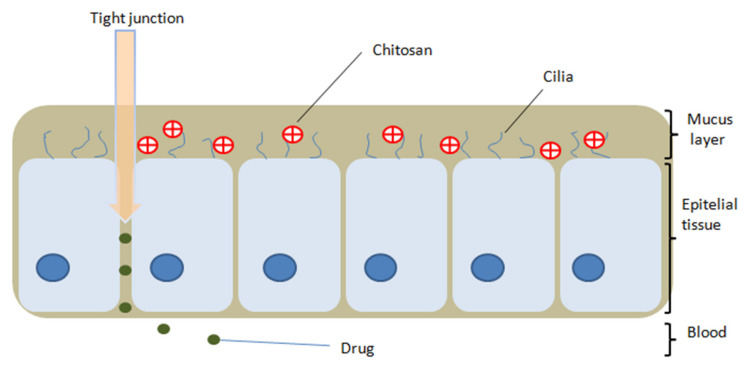
Mucoadhesive property of chitosan on the nasal mucosa and opening of the epithelial tight junction effect.

Chitosan has the property of being able to adhere to biological surfaces due to electrostatic interactions and hydrogen and hydrophobic bonds formed between the functional groups of chitosan and mucosal molecules [6,31,54]. In an acidic environment, chitosan amino groups charge positively [31] and can thus interact with the negatively charged mucin molecules from the mucous membranes, resulting in mucoadhesive properties (Figure 2) and favoring drug release from the matrix [8]. Initially, the polymer interacts with the mucosa, and is then deposited on the membrane [35]. Chitosan inhibits mucociliary development and differentiation by increasing factor TGF-b1 and interferes with the formation of tight junctions in nasal respiratory epithelial cells [55,56].

The mucoadhesive property of the polymer is directly proportional to the size of the chain and its flexibility; the more flexible the chain is, the easier it is to form a network with the mucus, increasing penetration [35].

**Figure 3:**
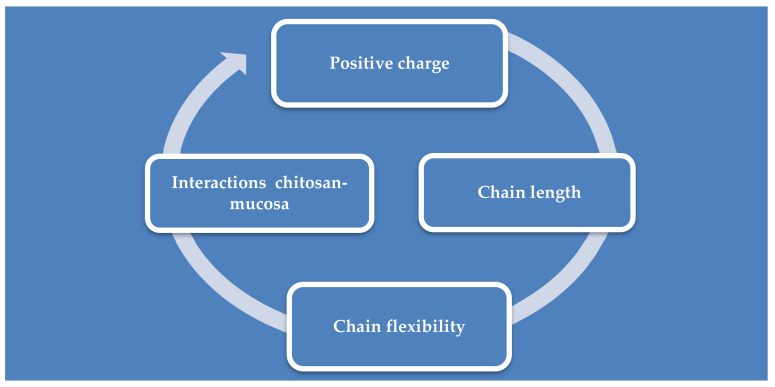
Factors influencing chitosan mucoadhesion.

The positive charge of chitosan is responsible for its application as a permeability enhancer [8]; the amino and hydroxyl groups form electrostatic interactions with sialic acid present in the nasal mucosa [9,57,58]. The amino groups bond ionically with sulfonic acid of the mucus and the amino and hydroxyl groups form hydrogen bonds with the mucus [35], leading to reversible opening of the tissue junction and the permeation of substances through the nasal epithelium is increased [59,60]. However, the permeation is also improved by prolonging the residence time on the nasal mucosa, due to the mucoadhesive properties of chitosan and, in this way, a better bioavailability is obtained [9,59] through a shorter latency and a faster absorption [61]. Most enhancers of penetration through the nasal mucosa cause mucosal irritation, but chitosan is considered a safe, non-irritating material, both locally and systemically, and is biodegradable [59]. The absorption of drugs from the nasal cavity and their transport to the brain or into the systemic circulation are influenced by mucociliary clearance. This is a physiological defense mechanism of the respiratory system which eliminates external substances by covering them with a layer of mucus, reducing the residence time of drugs administered intranasally [62]. However, the mucoadhesive property of chitosan is also responsible for the decrease of mucociliary clearance, because it adheres to the nasal mucosa and inhibits mucociliary development and differentiation by increasing the factor TGF-b1 [55,56]. The degree of this decrease is also influenced by the viscosity of the formulations [59]. The factors that influence the adhesion of chitosan on mucous membranes are summarized in Figure 3.

An in vitro study on nasal respiratory epithelial cells showed that cell differentiation took place on the control sample and the cells had a polygonal morphology, whereas the morphology of the cells in the chitosan sample was irregular, without cilia formation. The results also showed that chitosan decreases mucus secretion, which blocks the access of pathogens through epithelial tissue by inhibiting mucus-secreting cells and ciliated cells [55].

The ability of chitosan to adhere to mucous membranes has been the subject of several studies. It has been shown that bovine serum albumin, incorporated in chitosan nanoparticles, was released for 6 days in conditions similar to intestinal fluid at pH = 7.5, so it can be concluded that chitosan is a polymer that is suitable for drug release when the mucous membranes are targeted [6].

### 2.2. Superficial Tension

Chitosan displays low surface activity, consistent with its structure, which does not have large hydrophobic groups that can be adsorbed at the air/solution interface [5,8]. The interfacial properties of chitosan on air/water and oil/water surfaces were analyzed, and it was demonstrated that chitosan does not exhibit surface activity with concentrations lower than 0.1%. A gradual decrease in the surface tension was recorded with the increase of the chitosan concentration, indicating an arrangement of the hydrophilic and hydrophobic groups at the oil/water interface [8].

The surface tension of chitosan in 1% acetic acid is higher than that in a pure solvent. In the case of chitosan in acetate buffer at a pH of about 6–6.5, a slight decrease in surface tension was observed due to the stronger hydrophobic character, and only a small number of amino groups were protonated [5,8]. An increase of the chitosan concentration in acetate buffer solution leads to a decrease of the surface tension by the partial adsorption of the hydrophobic parts of the chitosan structure at the air/solution interface. As the pH decreases, there is a greater decrease in surface tension, because at an acidic pH, the amino groups receive a positively charged hydrogen atom, giving chitosan a more pronounced hydrophilic character [5].

The low-molecular-weight chitosan hydrochloride, a chitosan derivative, displays high surface activity, with researchers indicating that it can form a film at the air/water interface, leading to a decrease in surface tension [5,8]. In the case of another derivative, *O*-carboxymethyl chitosan, with increasing concentrations to 0.05 mg/mL, there was a decrease in surface tension; a higher concentration value then no longer influenced the surface tension [5].

### 2.3. Zeta Potential

Investigators have studied the surface charge and partial cross-linking of chitosan. The average value of the zeta potential for pure chitosan was shown to be −25.8 ± 3.74 mV, which resulted in a large surface charge, and this value decreased after cross-linking. The small difference between the values of the zeta potential before and after cross-linking led to the conclusion that the cross-linking was not total, but only partial, so it is possible to obtain additional interactions that help the incorporation of drugs [63].

Shun-Hsien Chang and collaborators made a connection between the zeta potential and pH. They performed a series of determinations on chitosan with different molecular weights (3.3–300 kDa), at pH 5, 6, and 7. For pH 5 and 6, the zeta potential values were positive, and higher for pH = 5. With an increase of the pH, the values decreased, even becoming negative at pH = 7 for chitosan with a molecular mass of 300, 156, and 72.1 kDa. This indicates that the lower the pH, the more positively the amino groups are charged. Obtaining these negative values can be explained by the formation of aggregates by intra- and intermolecular hydrogen bonds, which decreases the number of available amino groups [64].

The molecular weight also increases the zeta potential because when the molecular weight is higher, more amino groups are available and able to receive a hydrogen atom [64].

The determinations made by Gartzindia O. et al. showed that the zeta potential of chitosan-coated nanostructured lipid carriers for intranasal administration was +28 mV, compared to uncoated nanostructured lipid carriers, where the value was −30.3 mV, thus indicating a good adsorption of chitosan on the nanostructured lipid carrier’s surface and an increase in the particle size by about 10 nm [65].

### 2.4. Interactions of Chitosan with Other Molecules

Interactions between polymers and molecules help develop drug delivery systems, and the type of connections provides information about the release of drugs from the system [66]. The interactions and types of bonds between chitosan and its derivatives with other molecules are summarized in Table 1.

#### 2.4.1. Proteins

Although protein-based drugs are used in the treatment of diabetes, dyslipidemia, cancer, and other diseases, they have many disadvantages, such as a low bioavailability, significant instability due to the aggregation and separation of proteases, and a short half-life in plasma. All of these issues have led to the development of controlled release systems. One of these systems is a chitosan-based encapsulation system for the delivery of proteins and peptides which employs the properties of chitosan as a starting point to increase the penetration of substances through tissues [36].

The conclusions of J.R. Azevedo et al. were that the adsorption of insulin on the surface of chitosan nanoparticles led to a change in enthalpy, indicating that part of the amount of insulin interacts with chitosan; enthalpy changes are due to conformational changes and hydrophobic and electrostatic interactions, but also hydrogen bonds [67]. Chitosan nanoparticles loaded with insulin, based on ionic bonds, increased absorption after intranasal administration to rabbits, and insulin was released in a biologically active form [67,68].

Thermodynamic parameters have indicated that the interactions between chitosan nanoparticles with trypsin and trypsin inhibitors are mostly based on van der Waals bonds and hydrogen bonds. The size of the polymer influences the stability of the chitosan-protein conjugate; the higher the degree of polymerization, the higher the stability of the polymer-protein compound [36].

Another study investigated the influence of chitosan on the secondary structure of proteins. The proteins used were bovine serum albumin (BSA), containing two residues of tryptophan, and human serum albumin (HSA), containing one residue of tryptophan. In the case of BSA, the bonds were hydrophobic, and for HSA, the bonds were electrostatic. However, these interactions between chitosan and protein led to conformational changes in the protein structure, so the *α*-helix decreased by 6% for both proteins after interactions with chitosan. This conformational change can be explained by the decrease in the intensity of BSA and HAS fluorescence in the tryptophan area after the binding of chitosan near the tryptophan residues in proteins, which influences the drug-protein binding [36].

#### 2.4.2. Lipids

In vivo animal studies have shown that positively charged chitosan binds to fatty acids, bile acids, and cholesterol in the stomach, where they form complexes that are absorbed in the small intestine and are then excreted from the body. It was concluded that the interactions between chitosan and lipids are electrostatic (between the amino groups of chitosan and the carboxyl groups of fatty acids) and hydrophobic, and that there are hydrogen bonds between the hydroxyl groups of cholesterol and chitosan. However, there is also the hypothesis that chitosan may contribute to changes in lipid conformation [5].

The electrostatic interactions between chitosan and the unilayered 1,2-dioleoyl-sn-glycero-3-phosphocholine membrane led to the conclusion that chitosan adsorption occurs at the membrane level [5].

Due to the interaction between the positive groups of chitosan at pH > 6.5 and the hydrophobic groups of oleic acid, chitosan has been used by researchers to stabilize a nasal W/O emulsion of oleic acid. The stability of the system is also based on electrostatic interactions between the protonated amino groups of chitosan and the carboxyl groups of oleic acid [69].

Studies have shown that chitosan coating influences the adsorption and in vivo resistance of liposomes, resulting in superior properties compared to uncoated liposomes in terms of drug delivery. The advantages of chitosan-coated liposomes loaded with rifampicin are as follows: A higher encapsulation capacity of rifampicin, a higher stability of the chitosan-coated liposomes after nebulization, and improved mucoadhesion due to chitosan. This results in a decrease of the toxicity to epithelial cells of polymer-uncoated liposomes loaded with rifampicin [5].

#### 2.4.3. Other

Ion exchange between chitosan and indomethacin led to an improved solubility of indomethacin released from the solid chitosan-indomethacin complex, but the interactions between the amino groups of chitosan and the carboxyl of indomethacin also contributed to the formation of the complex [68].

A prolonged release of the drug from formulations can be obtained by the interaction of chitosan with negatively charged anti-inflammatory drugs (salicylic acid, ibuprofen, ketoprofen, and indomethacin) [72].

Through the interaction of chitosan with pyrazole-4-carbaldehyde, a Shiff base is obtained, which has a more pronounced antibacterial and antifungal effect than pure chitosan, exerting a synergistic effect [70].

Chitosan can form Shiff bases with different compounds, with various applications. Through the interaction of chitosan protonated with benzaldehyde-Pluronic-CHO, a Shiff base is obtained, which forms a hydrogel with a stretching capacity close to that of the joints. A Shiff base is formed between PEG and carboxymethyl chitosan, resulting in a hemostatic material to stop the bleeding caused by cuts. The Schiff base obtained from *N*,*O*-carboxymethyl chitosan and oxidized hyaluronic acid leads to the formation of an injectable hydrogel to prevent postoperative adhesion [71].

### 2.5. Antibacterial and Antifungal Action

Studies have shown that chitosan has antibacterial and antifungal properties. The antimicrobial action of chitosan depends on several factors: The positive charge of chitosan, the degree of *N*-deacetylation, the degree of polymerization [73], the molecular weight [74], the concentration [9], the pH [66], and the physical form [75]. The surface tension of polymers provides information about the antibacterial action and their stability, because ionic interactions between the polymer and the bacterium can be established. Bacteria have a negatively charged surface that interacts with the positive groups of chitosan. The integrity of the bacterial membrane is then altered and their development is diminished [63].

Chitosan inhibits the growth of bacterial cells by the following mechanisms:

- positively charged amino groups of chitosan interact with phospholipids and negatively charged proteins in the bacterial membrane [76], developing a layer on the bacterial cell surface and thus preventing the transfer of nutrients and blocking cell feeding [9];
- chitosan blocks the transcription of RNA from DNA by adsorption of chitosan infiltrated into DNA molecules [73];
- chitosan with low molecular weight penetrates inside the cell and interacts with negatively charged molecules in the cell, destroys its physiological activity, leading to cell death [9].

Sofien Benltoufa et al. determined the antibacterial properties of chitosan by soaking cotton with chitosan hydrogel and applying the material on bacterial cultures. The results showed that the antimicrobial activity of the chitosan-soaked material was better on gram-positive bacteria (*Listeria monocytogene* and *Staphyloccocus aureus*) than that of gram-negative bacteria (*Escherichia coli*) [73].

The antimicrobial action of chitosan is influenced by its molecular weight, but also depends on the type of bacteria. The main antibacterial mechanism of chitosan is thought to occur when positively charged amino groups of chitosan form electrostatic bonds with anionic molecules on the surface of bacterial cells, penetrate the cell, and interfere with the cell’s internal metabolism and kill it [74]. At a lower molecular weight, the antibacterial activity is more intense, destroying a higher percentage of bacterial cells compared to the antibacterial activity of chitosan with a higher molecular weight [6]. The results obtained by Pam et al. show that at a molecular weight of 97–123 kDa, the antibacterial activity on *E. coli* and *S. aureus* reached the maximum, and for *Candida albicans*, more intense activity was observed at 138 and 49.5 kDa [74]. There are studies showing that chitosan enters the cytoplasm of *E. coli* and enlarges the interstitial space in the membrane, leading to cell death, and also weakens or even breaks the membranes of *S. aureus* cells. Therefore, it was concluded that chitosan inhibits the development of gram-positive and gram-negative bacteria [9].

A study was performed to determine the synergistic effect of antibacterial substances and chitosan. The results of the determinations made on the film based on chitosan and ɛ-polylysine to evaluate the antimicrobial action on *E. coli*, *Bacillus subtilis*, and yeast showed that the diameter of the cultures decreased in all microorganisms, regardless of the ratio between the two, indicating the antimicrobial properties of both substances [77].

The antimicrobial action of the chitosan-molybdenum disulfate complex (CS-MoS2) was analyzed and a synergistic action of the two components was observed. The killing rate of *C. aureus* cells was the maximum and for *E. coli*, was 98.1% after a treatment with 10 µg/mL of CS-MoS2. Through the interaction of the positively charged complex with the negative groups of the cell membranes, degradation of the membrane and the cellular components occurred, decreasing the development of the bacterial cells [78].

A study that aimed to determine the antimicrobial action of Poly (vinyl alcohol) (PVA)/chitosan/*Bidens pilosa* (antibacterial plant) nanofibers showed a 55.6% inhibition of bacterial growth for *E. coli* and 40% for *S. aureus* in the case of pure chitosan compared to the PVA. The results obtained for the *Bidens pilosa* extract and PVA/*Bidens pilosa* extract/chitosan nanofibers indicate more inhibiting activity in the case of *E. coli* (by 11.4%) and *S. aureus* cells (by 39.3%) than a crude extract [79].

### 2.6. Immunostimulatory Effect

Recent data indicate that chitosan stimulates the immune system and increases immunocompetence [80]. It can activate macrophages and natural killer cells, attack tumor cells, amplify the activity of B/T lymphocytes, and strengthen cellular and humoral immunity [17]. Tests have shown an increase in IL-β1 and IL-2, thus increasing immunity [9].

The replacement of the primary amino group from C2 of chitosan with a quaternary amino group leads to the development of electrostatic bonds between the formed derivative and the sialic acid in the mucosa, improving the mucoadhesion, but also the immunostimulatory effect [34].

After the intragastrical administration of hydroxybutyl chitosan to mice for 21 days, the phagocytic activity of the macrophages was assessed by a carbon sequestration test, and it was concluded that their ability to phagocytose increased proportional to the hydroxybutyl chitosan concentration. This indicates a stimulation of the immune system due to the activation of the complementary system mediated by hydroxybutyl chitosan [17].

The immunostimulatory effect of chitosan was also determined by an evaluation of cytokines, in which the emulsion with recombinant tetravalent dengue antigen containing chitosan as a stabilizer had a good ability to activate IL-12 and IL-1β compared to the control sample. Therefore, the action of stimulating the immunity of the emulsion with chitosan was confirmed, in addition to the release of the antigen [69].

Lymphocyte proliferation was tested at different concentrations of hydroxybutyl chitosan and at different times. At a concentration greater than 100 µg/mL hydroxybutyl chitosan, an increase in lymphocyte proliferation was observed in vitro. It was also tested in vivo in mice by administering hydroxybutyl chitosan for 14 days. At doses higher than 100 mg/kg/day, the highest proliferation was established, showing that the chitosan derivative can improve the synergistic effects of the immune response by improving the overall immunocompetence [17].

Tests including mice have shown that chitosan-based nanoparticles improve their immune response against Brucellosis [81].

### 2.7. Antitumor Action

Chitosan has been shown to have cytotoxicity and an antiproliferative effect on cancer cells. Research has demonstrated an inversely proportional relationship between the cytotoxic action and the molecular mass of chitosan [9]. Other studies on antitumoral drugs have revealed that, when coated with a layer of chitosan, an increase in the cytotoxic effect compared to the substances as such was observed [31].

Analyses performed on mice with liver tumors showed that an oral administration of aqueous chitosan solution led to a decreased tumor volume [9].

One study tested the action of the antitumor agent casiopein III-ia, administered alone or incorporated into chitosan nanoparticles. The results showed that the survival rate of mice transplanted with B16 melanoma cells, which were given chitosan nanoparticles loaded with cassiopein, was higher compared to those which received the drug as such. This was achieved due to a longer residence time at the site of action, but also thanks to the pKa of chitosan, because the release of the substance from the chitosan nanoparticles was achieved at an acidic pH of the tumor [82].

### 2.8. Hemostatic Effect

The procoagulant mechanism of chitosan is not yet well-established, but most studies have concluded that positively charged amino groups of chitosan interact with negatively charged blood thrombocytes [83] and erythrocytes [83,84], leading to red blood cell agglutination and the stopping of bleeding [85]. In vitro studies have shown that chitosan induces blood clotting, even when heparin is present in the blood, indicating that its hemostatic action is independent of clotting factors [42].

In contrast to chitosan, its oligomer, chitosan oligosaccharide, has been studied for its effect on human blood. The results indicated that it prolongs the activated partial thromboplastin time, leading to anticoagulant action [84].

Chitosan with a higher molecular weight contains a larger number of amino groups and the hemostatic effect is thus improved [85]. Its combination with other hemostatic substances can increase the clotting capacity, achieving a synergistic hemostatic effect [83]. 

A disadvantage of chitosan is its insolubility in water, when its hemostatic action can be negatively influenced in a non-acidic environment [85].

### 2.9. Antioxidant Action

The antioxidant action of chitosan is explained by its ability, proven in vitro, to capture oxygen free radicals [9]. This action is due to free amino groups that have the ability to scavenge free radicals. Its antioxidant ability decreases with a decreasing number of amino groups, through chemical changes of chitosan [83].

The antioxidant activity of chitosan and aminated chitosan was evaluated by a spectrophotometric method, using ABTS (2,2’-azinobis-(3-ethylbenzothiazoline-6-sulfonic acid)). A decrease of the intensity of the bluish-green color of ABTS **^· +^** represents the acceptance of an electron from the antioxidant substance. In the case of the two polymers, it was determined that aminated chitosan has a greater ability to scavenge free radicals than chitosan [86].

The ability to capture hydroxyl radicals by chitosan and chitosan derivatives with propane sulfonated groups (propane sulfonated chitosan and dipropane sulfonated chitosan) was tested and compared to vitamin C. The strongest antioxidant effect was exhibited by dipropane sulfonated chitosan, followed by propane sulfonated chitosan, vitamin C, and chitosan. The ability to scavenge the superoxide radical was similar for the three polymers, with the best ability being shown by dipropane sulfonated chitosan, followed by propane sulfonated chitosan and chitosan. Deference is shown in the case of vitamin C, which had the highest capacity to capture superoxide radicals. In both cases, the antioxidant action was enhanced by increasing the polymer concentration [87].

The association of chitosan with other antioxidants is a real interest in current research. An in vivo study on aged mice investigated the antioxidant action of chitosan, collagen, and the collagen/chitosan complex on superoxide dismutase and malonic dialdehyde. The test results showed that the collagen/chitosan complex had a greater ability to reduce the concentration of malonic dialdehyde and increase the activity of superoxide dismutase; in conclusion, when combining the two substances, a synergistic effect is obtained [88]. The same synergistic effect is obtained by linking chitosan with amino acids, which have their own free amino groups and thus increase the ability to clean free radicals [83].

The antioxidant action of nasal chitosan-coated resveratrol microparticles is enhanced by the polymer’s ability to open the tight epithelial junction in the nasal cavity, promoting the absorption of resveratrol in the brain [89].

## 3. The Nasal Route

Nasal drug administration is an increasingly interesting topic for research and development in the pharmaceutical field [4,90]. The nasal cavity has an area of approximately 150–200 cm^2^ [91], is well-vascularized, and exhibits very good absorption [92], allowing multiple opportunities for the formulation of drug delivery systems to bypass the blood–brain barrier, such as gels [93], nanoparticles [94], microparticles [95], microspheres [96,97], emulsions [98], powders [99,100], lipid nanostructures [101], and inserts [102].

**Figure 4:**
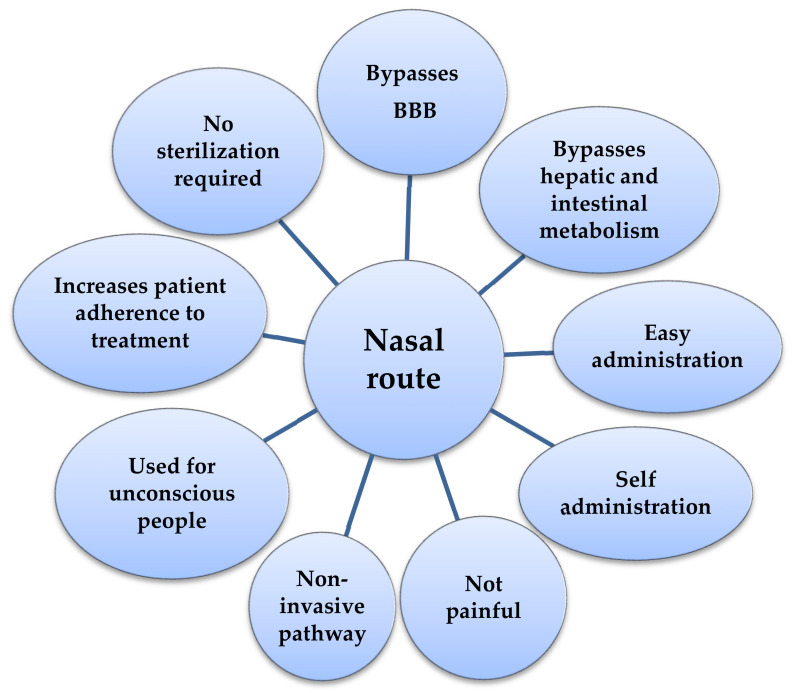
The benefits of nasal administration.

The active substances reach the brain directly from the nasal cavity, along the trigeminal nerve and through the olfactory system, bypassing the BBB in a non-invasive way [9,103], or are absorbed through the nasal epithelium into the systemic circulation and then pass by the BBB in the central nervous system (CNS) [104]. Systems that deliver the drug without being invasive and without penetrating BBB are considered to be third-generation systems [11]. Most of the advantages of using the nasal pathway are depicted in Figure 4.

The nasal route has a higher bioavailability than the oral route, avoiding hepatic [9] and intestinal metabolism [105]. This route can be used for any age category, but especially in children and the elderly or unconscious people [3,4,103], without the need for qualified staff [9,61]. Nasal administration has other advantages: It is not painful [60], it does not require sterilization [9], it is non-invasive [61], parenteral administration can be avoided (e.g., for insulin) [3,4,103], and it increases the patient’s adhesion to treatment [9]. Enzymatic and acidic degradation in the gastrointestinal tract is prevented by the nasal administration of drugs (e.g., insulin) [106] and the effect of systemic dilution is minimized [104].

Like any route of administration, the intranasal route has some disadvantages and restrictions: Differences between the nasal cavity of animals and humans [91]; mucociliary clearance; the enzymatic degradation of drugs [107]; the short residence time of drugs in the nasal cavity; unsatisfactory absorption through the nasal epithelium [9]; the bioavailability is relatively low [108]; and after nasal administration, the air flow is limited [109]. The most common adverse reaction after intranasal administration is mucosal irritation. Many of these disadvantages are overcome by formulating chitosan-based drug delivery systems, due to their properties, previously presented in this paper: Biocompatibility, biodegradability, non-toxicity, mucoadhesion, and permeability enhancer role [108]. There are also issues related to the safety of formulations based on chitosan and its derivatives that need to be investigated, such as the toxicity of the pharmaceutical forms, drug release, the stability of the systems [31], and the safety of the long-term administration of nasal drugs and vaccine delivery systems [62].

## 4. Chitosan in the Development of Intranasal Vaccines

In the case of vaccines, a vehicle is needed to deliver the antigen and, preferably, to have a nonspecific immunostimulatory effect, which improves the efficiency of immunization [34]. To increase the immune response of vaccines, adjuvants are introduced into their component, increasing the potency and efficiency of immunization. The adjuvants used are generally biodegradable, non-toxic, and biocompatible, and have their own immunostimulatory capacity. These include chitosan and its derivatives, hyaluronic acid, and sodium alginate [18,100].

Chitosan is one of the compounds that meets these criteria and has been approved as an adjunct in the formulation of human and veterinary vaccines [34,110]. By administering vaccines using chitosan-based systems, antigen degradation is prevented, cell absorption (cellular uptake) is increased, and a superior immune response is obtained [111]. The immunological activity of chitosan and the effect of the chitosan-based vaccine are influenced by the molecular weight and the degree of deacetylation [34,111]. Chitosan with a higher molecular weight forms more bonds with the antigen, thus obtaining a more stable system and a more efficient immunization. Additionally, a higher degree of deacetylation influences the immune response favorably [111]. Through electrostatic interaction, a close connection can be formed between the protonated chitosan and the antigen, thus increasing the immune response. The potency of immunoglobulins that neutralize the virus was increased when Diphtheria or Norovirus antigens were administered when chitosan was used as an adjuvant [34].

Chitosan derivatives take over the properties of chitosan, but also display some improvements, such as solubility, or in the case of *N*-trimethyl chitosan, a superior immunostimulatory effect [111]. Studies have been accomplished using *N*-trimethyl chitosan as a delivery system for nasal influenza vaccine subunits [81].

Chitosan nanoparticles loaded with the recombinant MxiH antigen of *Shigella flexneri* (a gram-negative bacterium, and the cause of shigellosis in humans) were tested on mice, intranasally, and an increase in IgG and IgA antibodies was obtained compared to the control group. The use of the polymer in antigen administration has led to an increase in cellular uptake from the nasal epithelium and M cells; thus, humoral and mucosal immunity is stimulated due to chitosan, which indicates that chitosan-based nasal vaccines have a superior immunogenic effect [112].

A comparison of the H5N1 nasal vaccine with and without chitosan as an adjuvant, administered to mice, concluded that the one with chitosan induces the appearance of a greater amount of antibodies against the influenza virus, which makes chitosan a good candidate as an adjuvant in the development of nasal vaccines [34].

Nasal vaccination against hepatitis B, with chitosan nanocapsules loaded with imiquimod containing TLR7, resulted in high levels of Ig G1 and IgG2a antibodies and specific long-term immunity [18]. For the administration of the hepatitis B antigen, modified PLGA chitosan microparticles with nasal administration were developed. One hour after administration, 32.6% of the microparticles were detected in the nasal cavity, concluding that chitosan can be used as an adjunct in the nasal delivery of vaccines, because long-term contact between the antigen and mucosa increases the vaccine absorption and effect. The *N*,*N*,*N*-trimethyl chitosan derivative is used in the formulation of intranasal vaccines [113]. Tests on *N*,*N*,*N*-trimethyl chitosan nanoparticles loaded with inactivated influenza virus, administered intranasally to mice, had the same results [34].

In the process of immunization against the influenza virus by the nasal route, the antigen, incorporated in chitosan-coated nanoparticles, crosses the epithelial barrier through microfold cells more easily, reaching the lymphoid tissue, associated with the nasal area, and dendritic cells more easily take over the nanoparticles. A previous study showed that chitosan is an immunoadjuvant because it obtained a superior immune response in mice that received chitosan-coated nanoparticles compared to the group that received uncoated nanoparticles [114].

Toxicity tests for nanoparticles used in nasal immunization based on *N*,*N*,*N*-trimethyl chitosan conjugated to ovalbumin showed that the toxicity in the nasal epithelium and cilia, in rats, is lower compared to other groups, indicating that the use of the polymer in nasal immunization is safer and more effective [34]. Therefore, it is a suitable polymer for antigen delivery [35].

## 5. Chitosan-Based Formulations for Intranasal Administration

Recently, nanoformulations for intranasal administration that prevent the degradation of molecules and increase their absorption in the brain have begun to be studied (Table 2). By coating the nanoformulations with chitosan, longer contact is obtained on the nasal mucosa, increasing the absorption [115].

### 5.1. Hydrogels

Chitosan-based hydrogels are able to transport peptides, proteins, growth factors, or other drugs directly to the site of action, reducing the risk of side effects [9]. A three-dimensional network of the gel can protect the easily degradable incorporated cells or substances, simultaneously ensuring a controlled release of the pharmaceutical ingredient from the formulation [116]. The gels have an emollient effect, reducing the irritation of the nasal mucosa. Due to the increased viscosity, the fraction of the swallowed substance decreases and a larger amount is thus available at the nasal level for cellular uptake [117].

The development of thermo-sensitive hydrogels, which are administered in the nasal route and which pass from the soil phase at room temperature to the gel phase when they reach body temperature is constantly advancing [3,103,116]. A hydrogel based on chitosan and β-glycerophosphate is a thermo-sensitive one [3,118]. This type of hydrogel is used for the nasal administration of doxepine, bypassing BBB [119]. Gelation is achieved by electrostatic attraction between positively charged amino groups of chitosan and anionic phosphate groups of β-glycerophosphate. In addition, hydrogen bonds and electrostatic interactions occur between chitosan chains [118]. Another example is chitosan hydrogel and hydroxypropylethyl cellulose for the nasal application of ropinirole. Its distribution in the brain was about 90%, which shows that the drug bypassed BHE, reaching from the nasal cavity directly into the brain [4].

Mucociliary clearance decreased after the intranasal administration of a chitosan hydrogel with insulin in rats, insulin release was slow, and plasma glucose levels decreased over 24 h, leading to once-daily insulin administration, without the need for invasive administration. Spectrophotometric analysis of insulin release from chitosan and PVA hydrogel, after intranasal administration, showed that glucose levels were maintained within 6 h [4,120]. Jeffrey et al. analyzed the distribution of insulin in the brain after the intranasal administration of fluorescein-isothiocyanate-insulin (FITC) to rats. The data showed that insulin can reach the brain through the perineural space of the trigeminal nerve and the cerebral perivascular spaces after administration through the nasal route, in the form of a biologically active substance, without suffering from any degradation, having a binding capacity and activating insulin receptors [61,108].

#### Hybrid Hydrogels

Hydrogels can incorporate nanoparticles or microparticles into their three-dimensional structures, producing hybrid hydrogels with improved mechanical properties, adhesion on tissues, and a controlled release of drugs. The release of vancomycin from vancomycin-HPMC nanoparticles incorporated into the chitosan/glycerophosphate hydrogel was prolonged, as the release was achieved by diffusion from the nanoparticles and then from the hydrogel [121].

Another example of a hybrid hydrogel for nasal administration contains chitosan nanoparticles loaded with levodopa and incorporated into the thermo-reversible hydrogel of Pluronic 127. This formulation increased the residence time due to the viscosity of the hydrogel, but also due to the mucoadhesive properties of chitosan, simultaneously decreasing the mucociliary clearance [122].

Toxicity tests performed on thermo-sensitive hydrogel based on Pluronic and loaded with chitosan microspheres with lorazepam did not indicate toxicity or irritation to the nasal mucosa [117].

### 5.2. Nanoparticles

The intranasal administration of polymeric nanoparticles containing various substances has several advantages, in addition to the general advantages of the nasal pathway: It prevents the enzymatic and chemical degradation of substances [106], increases the stability of volatile drugs, increases the residence time of substances at nasal mucosa, and has a better bioavailability [6,114]. The most commonly used mechanism for obtaining nanoparticles with the active substance is by introducing the active molecules during the nanoparticle manufacturing process, when the molecules are better fixed in the nanoparticle matrix. Less frequently used is the method of introducing substances after obtaining nanoparticles [6]. Nanoparticles have a high capacity to encapsulate substances [106] and a large contact surface to interact with substances, but also with mucous membranes [81].

The hydrophilic groups (hydroxyl and amino) of chitosan and its derivatives allow non-covalent binding to mucous membranes or epithelial tissue [127]. The bioadhesive properties of chitosan can increase the residence time of nanoparticles loaded with various drugs in the nasal cavity and increase their absorption. Chitosan nanoparticles loaded with bromocriptine, for Parkinson’s disease, can cross the nasal mucosa, preventing degradation of the substance. Studies have shown that mice treated with such a bromocriptine delivery system had a higher exercise capacity than those treated with bromocriprine solutions [128].

Rotigotine, a drug less commonly used in Parkinson’s disease due to its rapid degradation, has been incorporated into chitosan nanoparticles for nasal administration. Bhattamisra et al. obtained a higher concentration of the substance in the brain, after the intranasal administration of chitosan nanoparticles in rats, compared to the nasal administration of rotigotine solution. In terms of the route of administration, the highest concentrations of substance were recorded after intranasal administration, followed by the intravascular route and the last one, per os [123].

Chitosan-coated rivastigmine nanoparticles were demonstrated to increase the concentration of rivastigmine in the brain after intranasal administration in Alzheimer’s disease. The results indicated a continuous release of rivastigmine over 24 h. The concentration of the substance in the brain was significantly higher after the intranasal administration of chitosan-coated nanoparticles compared to the intravascular or intranasal administration of rivastigmine solution [128].

Chitosan nanoparticles administered in the nasal pathway have also been investigated for Alzheimer’s disease; loading them with various active moieties, such as insulin [129], galantamine (where a decrease in acetylcholinesterase is observed), and piperine (a potential neuroprotective compound in Alzheimer’s) resulted in a continuous release from the nanoparticles [128]. Chitosan-based nanoparticles for intranasal administration have also been investigated for other diseases, including spinal heart injuries (methylprednisone) and cerebral ischemia (rutine) [129].

### 5.3. Microparticles

Stearic acid-based microparticles loaded with resveratrol and coated with chitosan have been developed. Resveratrol is a natural polyphenol that is found in fruits, vegetables, and red wine and has anti-inflammatory and antioxidant actions. A comparative study was conducted after the intranasal administration of 0.2 mg of resveratrol to rats from four different formulations. The best result was obtained after the administration of an aqueous suspension of microparticles loaded with resveratrol and coated with chitosan, when the maximum concentration detected after 60 min was approximately 9.7 µm/mL after nasal administration, followed by chitosan hydrochloride suspension with resveratrol (approx. 1.3 µm/mL), resveratrol microparticles not coated with chitosan (~0.79 µm/mL), and aqueous resveratrol suspension. This result can be explained by the ability of chitosan to reversibly open the tight epithelial junction in the nasal cavity, increasing the absorption of resveratrol, but also increasing the residence time due to mucoadhesive properties [89].

### 5.4. Powders and Microspheres

The nasal route is used for powders with poorly soluble substances. Powders for nasal administration contain as little excipient as possible in the case of substances of low potency; for potent substances, where the dose is less than 5 mg, excipients for filling or transport are required. The dose for one nostril is about 10 to 25 mg of powder. Chitosan is used as an excipient for nasal powders with a filling role, to increase the residence time of the substance in the nasal cavity due to the mucoadhesive properties, thus increasing the absorption [130].

After the intranasal spray-dry of zolmitriptan powder in rats, containing chitosan as an excipient, it was determined that the delivery of the substance to the brain was similar to intravenous administration and improved, compared to intranasal administration of the suspension of the substance. Transport from the nasal cavity to the brain was made easier due to the ability of chitosan to open the tight junction of the nasal mucosa, facilitating distribution in the brain [130].

Microspheres can form a system of continuous drug release; they also protect drugs against enzyme degradation [117]. They can be obtained by spray drying, double emulsification, or complexation with other substances [131]. Microspheres become hydrated after absorbing water from epithelial cells, when the cells are reversibly dehydrated and in this way, promote junction opening and drug absorption [124,132].

Chitosan microspheres loaded with carvedilol, administered as dry powder to rabbits intranasally, have a bioavailability equal to approximately 70% of that intravenously [130].

In vivo studies on mice have shown that, after the intranasal administration of chitosan microspheres loaded with diltiazem, an effective dose, five times lower than in oral treatments, can be used [124].

### 5.5. Emulsions

The dispersed phase of nanoemulsions occurs in the form of droplets with a size of less than 200 nm, which presumes a large contact surface between the mucosa and the drops containing the drug [126,133]. The addition of chitosan in emulsions increases its residence time in the nasal cavity and improves contact with the mucosa; the physical stability of the emulsion is also better [125]. Nanoemulsions are formed by applying external shear stresses, and microemulsions are obtained by a thermodynamic process, which leads to a kinetic stability for nanoemulsions and a thermodynamic one for microemulsions [98,133].

Chitosan microemulsion with rivastigmine for intranasal administration has a high potential for distribution in the brain due to mucoadhesive properties, an increased viscosity, and a high contact angle [117].

In the case of cabergoline loaded in a chitosan-coated microemulsion and administered intranasally to rats, the substance reached from the nasal cavity directly into the brain, where a higher amount of drug was determined compared to the uncoated microemulsion administered intranasally and intravenously. Additionally, a higher absorption ratio in the brain/blood was recorded [134].

Rosmarinic acid is a natural compound with anti-inflammatory and antioxidant actions. It is also being researched for its neuroprotective effect. Attempts are being made to develop an intranasal formulation to avoid systemic side effects, hepatic metabolism, and the gastrointestinal tract. By evaluating the permeability of rosmarinic acid nanoemulsions, coated or non-coated with chitosan, through porcine nasal mucosa, it was concluded that a higher amount of rosmarinic acid was retained in the nasal mucosa after 8 h for the chitosan-coated nanoemulsion. This led to the conclusion that the polymer interacts with the components of the nasal mucosa, having a high penetration potential and presenting the possibility to obtain a slow release and a long time of action [125].

In vitro studies have indicated that coating a rotigotine nanoemulsion with chitosan for nasal administration resulted in a prolonged release and absorption of the substance through the nasal mucosa of approximately 85% in 4 h, compared to an absorption of approximately 65% obtained after testing the uncovered nanoemulsion, due to the mucoadhesive and the permeability enhancer effect of chitosan [126].

A nanoemulsion with quetiapine administered intranasally has a shorter action time compared to intravascular administration. The same results were observed for the nanoemulsion with zomatriptan [106]. Intranasal administration of the chitosan-coated olanzapine nanoemulsion resulted in a higher amount of substance in the brain [134].

### 5.6. Nano-Lipid Carriers

Nano-lipid carriers (NLC) are lipid-based drug delivery systems with an increased stability compared to solid lipid nanoparticles [135]. Lipid carrier nanostructures have a high capacity to incorporate drugs, have a low water content, and have the ability to incorporate molecules very well during storage, avoiding the premature release of drugs [117,136]. Chitosan-coated nanostructured lipid carriers improve drug absorption and their contact time with the mucosa [135].

A study on chitosan-coated NLC loaded with near-infrared dye showed the efficiency of distribution in the brain after intranasal administration, due to the mucoadhesive properties of chitosan and due to decreased mucociliary clearance, allowing the drug to reach the olfactory area. Chitosan augments the residence time of nasal pharmaceutical forms in the olfactory region. Moreover, by opening the epithelial junction in the nasal cavity, chitosan increases the transport of drugs through the epithelial mucosa [62].

## 6. Conclusions

Studying the properties of chitosan and its derivatives is necessary in order to evaluate the influence that they have in the development of formulations with nasal administration. Studies to date have shown that chitosan is biodegradable and biocompatible and has very good mucoadhesive properties, which makes it suitable for mucosal administration, leading to the formulation of nasal drug delivery systems. This route of administration is under continuous development, and studies are being undertaken to achieve the delivery of drugs with both local and systemic activity, especially for those targeting the brain.

Intranasal drug and vaccine delivery systems based on chitosan and its derivatives aim to increase the bioavailability of the pharmaceutical ingredients and to enhance the patient’s adherence, providing new treatment opportunities. Studies have been conducted for diseases such as Alzheimer’s disease, Parkinson’s disease, diabetes, depression, cerebral ischemia, cognitive impairment, communicable diseases, and others. These systems are of interest in the medical and pharmaceutical fields because they can include degradable drugs and allow the use of lower effective doses.

Nasal vaccine variants can be used to overcome the drawbacks of conventional injectable vaccines. These can prevent outbreaks or pandemics, such as the current one, leading to a more efficient management of communicable diseases. Using nasal vaccines can increase the percentage of immunized people. As the nasal route is non-invasive and fast, the vaccine can be easily administered at home, without the need for qualified medical staff and the person to go to the doctor.

Given the antibacterial, antifungal, immunostimulatory, antioxidant, hemostatic, and antitumor properties of chitosan and its derivatives, they can be used in combination with other molecules with the same effect, and obtain a synergistic effect.

Like any new pharmaceutical form developed, there are concerns about the toxicity and safety of the long-term use of nasal formulations and the stability of these systems.

## Figures and Tables

**Table 1:** Interactions of Chitosan and its Derivatives.

No.	Polymer	Molecule	Binding Type	Outcomes	**Index**
1	Chitosan	Insulin	Hydrophobic interactions Electrostatic interactions Hydrogen bonds	Conformational alteration	[67]
2		Insulin	Ionic bonds	Increased absorption	[68]
3		Trypsin and trypsin inhibitors	Van der Waals bonds Hydrogen bonds	Improves stability	[36]
4		HSA	Electrostatic interactions	Conformational changes	[36]
5		BSA	Hydrophobic interactions	Conformational modification	[36]
6		Lipids	Electrostatic interactions Hydrophobic interactions Hydrogen bonds	Improves system stability	[5]
7		Indomethacin	Ionic bonds Electrostatic interactions	Increase solubility	[68]
8		Pyrazole-4-carbaldehyde	Substitution reaction	Shiff base with superior antibacterial and antifungal effects	[70]
9		Pluronic-CHO-benzaldehyde	Substitution reaction	Schiff Base; hydrogel with joint-like properties	[71]
10	Carboxymethyl chitosan	PEG	Substitution reaction	Schiff Base; hemostatic material	[71]
11	*N*,*O*-carboxymethyl chitosan	Oxidized hyaluronic acid	Substitution reaction	Schiff Base: hydrogel that prevents postoperative adhesion	[71]

**Table 2:** List of some drug delivery systems for nasal administration based on chitosan.

Drug Delivery Systems	Content of Chitosan (% *w/v*)	Drug	Results	References
Hydrogel	2	Doxepin	The residence time in the nasal cavity Is increased and the drug bypass the blood brain barrier.	[119]
Hydrogel	1; 2; 3; 4; 5	Insulin	Decrease the mucociliary clearance leading to a slow release; insulin didn’t suffer any degradation during transport to the brain.	[120]
Nanoparticle	0.05	Rotigotine	Increase bioavailability and a higher concentration in the brain is achieved.	[123]
Microparticle	1.75	Resveratrol	The absorption of the drug is increased by opening the tight epithelial junction and enhanced the contact time.	[89]
Microsphere	2	Diltiazem	The effective dose required is less than the oral dose.	[124]
Emulsion	0.1; 0.3; 0.5	Rosmarinic acid	The penetration capacity is higher, and the release is slow.	[125]
Emulsion	1	Rotigotine	Prolonged release and increased absorption.	[126]
